# Circulating Tumor Cells Expressing the Prostate Specific Membrane Antigen (PSMA) Indicate Worse Outcome in Primary, Non-Metastatic Triple-Negative Breast Cancer

**DOI:** 10.3389/fonc.2020.01658

**Published:** 2020-09-03

**Authors:** Sabine Kasimir-Bauer, Corinna Keup, Oliver Hoffmann, Siegfried Hauch, Rainer Kimmig, Ann-Kathrin Bittner

**Affiliations:** ^1^Department of Gynecology and Obstetrics, University Hospital of Essen, Essen, Germany; ^2^QIAGEN GmbH, Hilden, Germany

**Keywords:** triple-negative breast cancer, circulating tumor cells, PSMA, androgen receptor, androgen receptor splice variant seven

## Abstract

**Background:** We analyzed mRNA profiles of prostate cancer related genes in circulating tumor cells (CTCs) of primary, non-metastatic triple-negative breast cancer (TNBC) patients (pts) before and after neoadjuvant chemotherapy to elucidate the potential of prostate cancer targets in this BC subgroup.

**Method:** Blood from 41 TNBC pts (*n* = 41 before / 26 after therapy) was analyzed for CTCs applying the AdnaTest EMT-2/Stem Cell Select. Multimarker RT-qPCR allowed the detection of the prostate specific antigen *PSA*, the prostate specific membrane antigen *PSMA*, full-length androgen receptor (*AR-FL*), and AR splice-variant seven (*AR-V7*).

**Results:** Before therapy, at least one prostate cancer related gene was detected in 15/41 pts (37%). Notably, in 73% of *AR-FL* positive cases, *AR-V7* was co-expressed. After therapy, CTCs of only one patient harbored prostate cancer related genes. *AR-V7*+ and *PSMA*+ CTCs significantly correlated with early relapse (*p* = 0.041; *p* = 0.00039) whereas *PSMA*+ CTCs also associated with a reduced OS (*p* = 0.0059). This correlation was confirmed for *PSMA*+ CTCs in univariate (PFS *p* = 0.002; OS *p* = 0.015), but not multivariate analysis.

**Conclusion:** Although CTCs that expressed prostate cancer related genes were eliminated by the given therapy, *PSMA*+ CTCs significantly identified pts at high risk for relapse. Furthermore, AR inhibition, often discussed for this BC subgroup, might not be successful in the primary setting of the disease since we identified *AR-FL*+ CTCs together with *AR-V7*+ CTCs, associated with therapeutic failure.

## Introduction

Triple-negative breast cancer (TNBC), accounting for 15–20% of all breast cancers (BC), has an destructive behavior which is associated with poor prognosis (1, 2). Neoadjuvant chemotherapy (NACT) is the standard of care (3, 4) and combination therapy containing carboplatin improved the pathological complete response (pCR) rate (5), as well as progression free survival (PFS) and overall survival (OS) in some clinical trials (6, 7). However, treatment options are limited since TNBC remains a biologically variable disease with different subtypes defined and thus, a target or signal transduction pathway for therapy is difficult to identify (8). Currently, immunotherapy is under investigation in this patient subset and has already shown a significantly improved pCR adding the checkpoint inhibitor anti-PD-1, pembrolizumab, to NACT in early TNBC with a trend seen for a prolonged event free survival (9).

Looking for new predictive biomarkers, prostate cancer related markers have been evaluated in TNBC for additional treatment options. In this context, based on findings in prostate cancer (PCA), the prostate specific membrane antigen (PSMA) has become an attractive molecular target for oncological imaging and radionuclide therapy using PSMA PET/CT in TNBC (10, 11). In addition, among the different subtypes defined (12), the luminal androgen receptor (LAR) subtype was found to be enriched in mRNA expression of androgen receptor (AR) and several downstream AR targets, resulting in enhanced sensitivity to the AR antagonist bicalutamide (13) which qualifies AR as a suitable target in LAR TNBC. In this context, ongoing clinical trials are testing the effectiveness of other AR inhibitors in TNBC, including abiraterone and enzalutamide, commonly prescribed in PCA (14–17). AR, overexpressed in 10–35% of TNBC, shows some similarities with the hormonal-receptors (HR) estrogen- (ER) and progesterone- (PR) receptor. AR is a member of the steroid-hormone-receptor family and functions after activation by binding of androgens as nuclear transcription factor. Similar to observations in ER-positive (+) BC, its expression has been associated with improved PFS and OS (8, 18, 19). In another retrospective trial, low AR expression was correlated with higher risk of distant metastasis, whereas high AR expression was correlated with prolonged survival. In addition, AR status was an independent predictor for better outcome regardless of tumor size, grade, and nodal stage (20). Further studies revealed that AR+ tumors were associated with small tumor size, lower histologic grade and stage (21). Interestingly, in the prospective German GeparTrio trial, pCR in TNBC after NACT was lower in AR+ compared with AR-negative disease. However, in accordance with other studies, AR+ tumors had a significant better PFS and OS as compared to tumors not expressing AR in the intention to treat population but stratified by subgroups these findings could only be shown for the TNBC patients. AR positivity selected a group with significant better PFS and OS in the non-pCR group, however, no difference with regard to AR expression was shown for the pCR group (22). In contrast, some other studies could not confirm these observations and have shown either no difference or worse outcomes for AR-positive (+) vs. AR-negative disease (23–27).

Comparable with data for HR, concordance of AR expression status between primary BC tissue and metastatic lesions was shown to be 15–35% (28). Consequently, AR expression on tissue samples might not be appropriate to select BC patients for AR-targeting drugs.

Therefore, a few metastatic BC studies have analyzed AR expression on circulating tumor cells (CTCs) in blood as a minimal invasive approach to assess the real time AR status (29–33). Most of these studies were performed in HR+/HER2- BC, but AR+ CTCs could be detected in 13% of metastatic TNBC cases applying mRNA expression profiling for CellSearch enriched CTCs (32) and in 91% of metastatic TNBC cases using the Maintrac Assay (30). Performing comprehensive molecular CTC characterization in early TNBC patients after immunomagnetic CTC-selection, we recently demonstrated that TNBC-derived CTCs appeared to upregulate most of the analyzed 17 transcripts or kept their expression frequency on a high level after therapy except for *AR* which was detected in 33% of the patients before but rarely after therapy (34). However, several studies on AR expression in patients with castration-resistant PCA demonstrated that not the AR full length (*AR-FL*) wildtype itself but AR splice variants, and in particular AR variant seven (*AR-V7*), have been linked to resistance toward anti-AR drugs like enzalutamide and abiraterone (35). In this context, *AR-V7*+ CTCs before AR blockade correlated with decreased PFS, decreased time on therapy and shorter OS as compared to patients without *AR-V7*+ CTCs (36, 37). In BC, the *AR-V7* variant was shown to be commonly expressed in primary BC tumor tissue and BC cancer cell lines, providing evidence to promote growth and mediate resistance to AR inhibitory treatment (38).

Based on the current findings, the complex interplay of AR and the prostate specific antigen (PSA) and PSMA (39) and the growing interest of the application of AR-targeted therapies in TNBC, we here analyzed mRNA profiles of CTCs for the expression of *AR-FL, AR-V7, PSA*, and *PSMA* in blood samples of 41 TNBC patients before and 26 TNBC patients after therapy to elucidate their prognostic value and their potential as therapeutic targets.

## Results

### Clinical Characteristics

The clinical characteristics of all patients evaluated before and after therapy are shown in Table 1. More than 50% of the patients were postmenopausal, the predominant histological subtype was ductal carcinoma and most of the patients had an aggressive tumor biology with a grade 3 tumor. The majority of the patients showed a Ki67 above 30% and presented with T1 and T2 tumors. At the time of primary diagnosis, two third of the patients were node-negative and except for two patients, all patients received NACT. The therapeutic regimens are shown in Table S1. Overall, response to therapy resulted in a ratio of 92% (46% pCR, 46% pPR) of responders and 8% of non-responders.

**Table 1:** Patient characteristics.

	**Total (% of all applicable)**
**Total**	41
Median Age (IQR) at diagnosis [years]	52 (15)
<50 years old	17/41 (41)
≥50 years old	24/41 (59)
**Menopausal Status**	
Premenopausal	8/41(20)
Perimenopausal	8/41 (20)
Postmenopausal	25/41 (60)
**Histology**	
Ductal	30/39 (77)
Lobular	1/39 (3)
Others	8/39 (20)
Not known	2/41
**Tumor Grading**	
I	0/41 (0)
II	11/41 (27)
III	30/41 (73)
Not known	0/41
**Ki 67**	
0–10%	2/37 (5)
11–30%	4/37 (11)
>30%	31/37 (84)
Not known	4/41
**Tumor Size at First Diagnosis (c/T)**	
T1a-c	16/41 (39)
T2	22/41 (54)
T3	3/41 (7)
T4	0/41 (0)
**Tumor Size After NACT (ypT)**	
ypT0	17/39 (44)
ypT1	12/39 (31)
ypT2	9/39 (23)
ypT3-4	1/39 (3)
Not applicable	2/41
**Nodal Status at First Diagnosis (c/pN)**	
Node negative	27/41 (66)
Node positive	13/41 (32)
N1	10/41 (24)
N2	1/41 (2)
N3	3/41 (7)
**Nodal Status After NACT (ypN)**	
Node negative	2/3 (66)
Node positive	1/3 (33)
ypN1	0/3 (0)
ypN2	1/3 (33)
ypN3	0/3 (0)
Not applicable	38/41
**Pathological Response**	
Complete response	18/39 (46)
Partial response	18/39 (46)
No response	3/39 (8)
Not applicable	2/41
**Chemotherapy**	
Yes	41/41 (100)
Neoadjuvant	39/41 (95)
Adjuvant	2/41 (5)

### Gene Expression Profiles in CTCs Before and After Therapy

**Figure 1:**
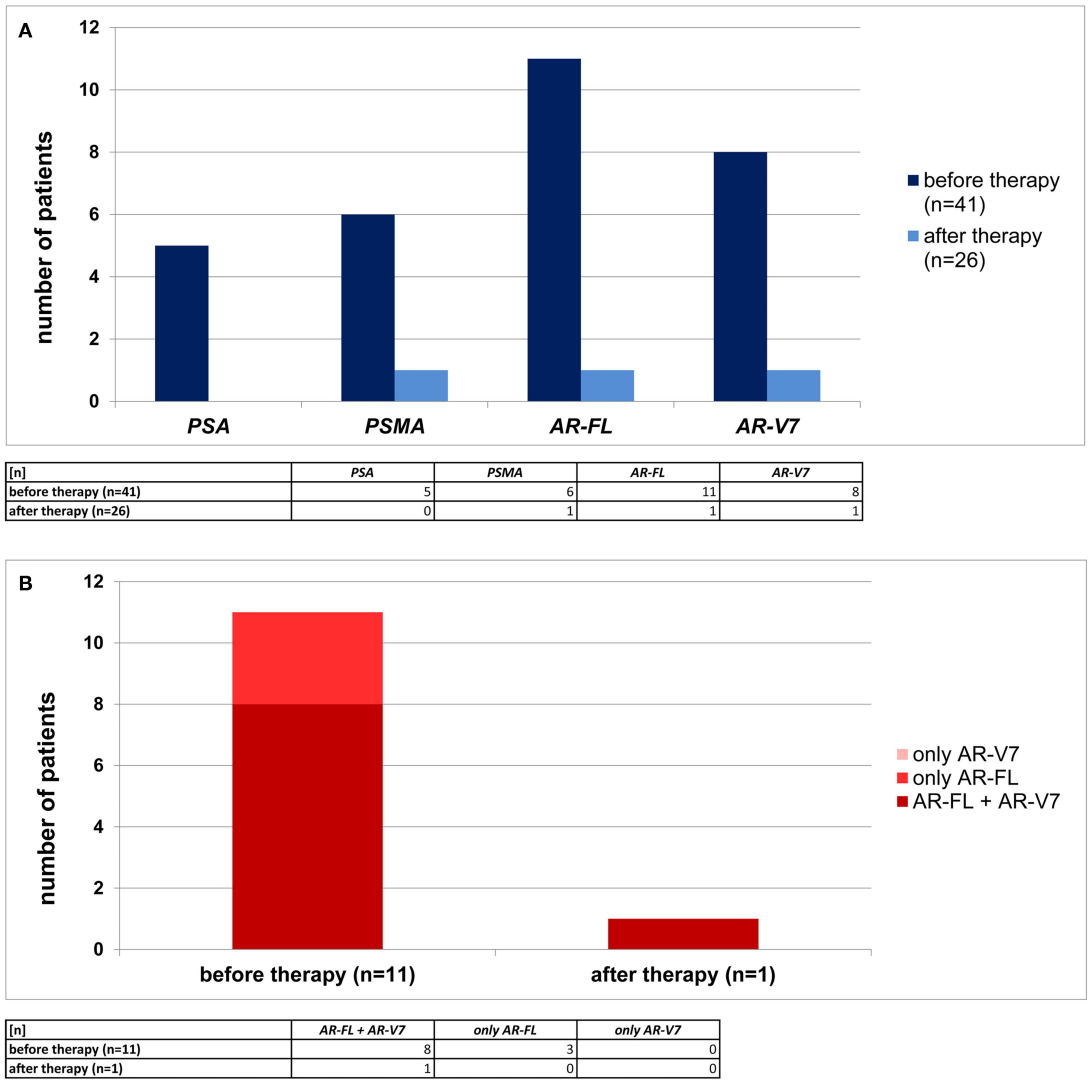
Prevalence of primary TNBC patients with prostate cancer related transcripts detected in CTCs. **(A)** In 41 TNBC patients before therapy (dark blue) and 26 TNBC patients after neoadjuvant therapy (light blue) *PSA, PSMA, AR-FL*, and *AR-V7* RNA profiles were examined. **(B)** Co-expression of *AR-FL* and *AR-V7* (dark red) was detected in the majority of *AR-FL*+ CTCs. Some patients displayed only *AR-FL*+ CTCs (red), but no patient was examined to have only *AR-V7*+ CTCs (light red).

In total, 41 primary, non-metastatic TNBC patients were analyzed for CTCs. Matched samples of these 41 patients were available after therapy in 26 cases resulting in 26 paired samples (before and after therapy). Using immunomagnetic selection *via* EpCAM, HER2, and EGFR, a patient was defined as CTC+ if overexpression of one of the four prostate cancer related genes was detected. Before therapy, at least one prostate cancer related gene was detected in 15/41 pts (37%). The expression of *AR-FL* was documented in 11/41 patients (27%), *AR-V7* in 8/41 patients (20%), *PSMA* in 6/41 patients (15%), and *PSA* in 5/41 patients (12%), respectively. Notably, as apparent from Figure 1B, in 8/11 patients (73%) of *AR-FL*+ cases before therapy, *AR-V7* was co-expressed. In 26 of the in total 41 patients analyzed before therapy, we were able to perform CTC analysis also after therapy. In only one of these 26 patients after therapy, we found CTCs with an overexpression of prostate cancer related genes (*AR-FL, AR-V7*, and *PSMA*, Figure 1A). In addition, this patient showed a persistence of *AR-FL* and *AR-V7* expressing CTCs and the presence of *PSMA*+ CTCs after therapy. In all the other 25/26 patients analyzed after therapy, no CTCs expressing prostate cancer related genes were detected.

Before therapy, *PSMA*+ CTCs were more often found in patients experiencing no pCR, as compared to those achieving one. Although these findings were not significant (two-tailed Fishers exact test: *p* = 0.19; Figure S2), the only patient harboring *PSMA*+ CTCs before therapy and achieving a pCR was the only patient in the pCR subgroup who deceased within the follow-up time.

### Survival Analysis

Thirteen relapses were documented after a median follow-up time of 16 months (range: 3–34 months). 8/41 (20%) of the patients died, eight of them BC specific, after a median survival time of 25 months (range: 3–38 months).

**Figure 2:**
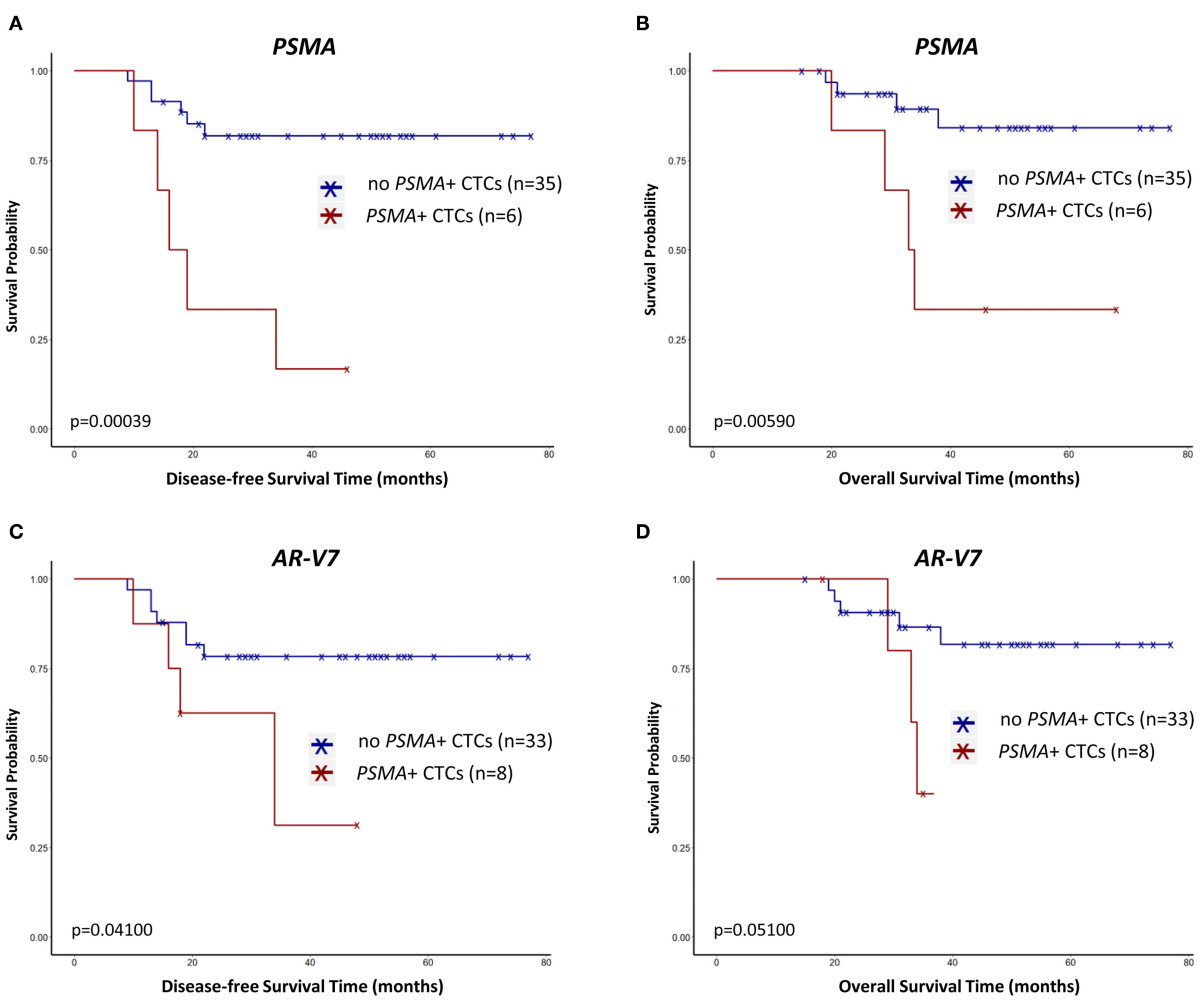
Survival curves regarding *PSMA*+ CTCs **(A,B)** and *AR-V7*+ CTCs **(C,B)** in primary TNBC patients before neoadjuvant treatment. Survival intervals were screened from the time of first diagnosis until the date of recurrence [here disease-free survival; (**A,C**)] or death [overall survival; (**B,D**)] and calculated with Kaplan-Meier estimator (Log-rank test).

*PSMA*+ CTCs (Figure 2A) and *AR-V7*+ CTCs (Figure 2C) before therapy significantly correlated with early relapse (*p* = 0.00039; *p* = 0.041). *PSMA*+ CTCs (Figure 2B) also associated with a reduced OS (*p* = 0.0059) while *AR-V7*+ CTCs (Figure 2D) reached borderline significance (*p* = 0.051). While half of the pts showing *PSMA*+ CTCs relapsed within 19 months after first diagnosis, more than half of the pts with *PSMA*- CTCs did not experience a relapse within the period of follow-up (Figure 2A).

**Figure 3:**
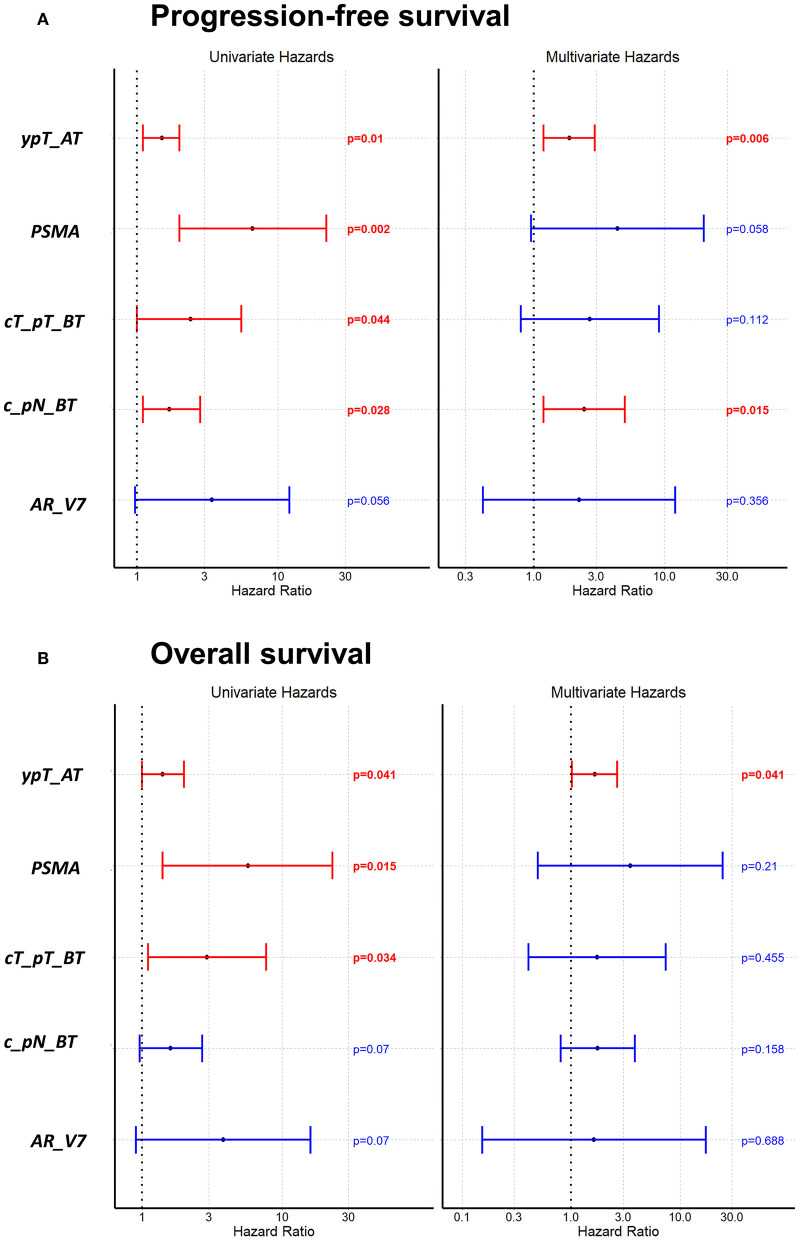
Univariate and multivariate Cox proportional hazard analysis regarding PFS **(A)** and OS **(B)**. Univariate analysis shows the prognostic value of *PSMA*+ CTCs for PFS and OS. ypT_AT: tumor size after therapy, cT_pT_BT: tumor size before therapy, c_pN_BT: lymph node status before therapy.

Figures 3A,B as well as Table S3 are showing survival analysis using Cox univariate and multivariate proportional hazard analysis with the standard staging parameters tumor size and lymph node involvement before and after therapy. In univariate analysis, *PSMA*+ CTCs turned out as a significant unfavorable predictor for PFS (Figure 3A; *p* = 0.002) and OS (Figure 3B; *p* = 0.015), respectively. Using multivariate Cox proportional hazard analysis, neither *PSMA*+ CTCs nor *AR-V7*+ CTCs, independently associated with a significant shorter PFS (Figure 3A) nor with OS (Figure 3B). With regard to clinical parameters, univariate analysis identified tumor size before and after therapy (*p* = 0.044; *p* = 0.0097) as well as lymph node involvement before therapy (*p* = 0.028) and non-pCR (*p* = 0.01) as unfavorable variables for PFS which was confirmed in multivariate analysis for non-pCR (*p* = 0.041) and lymph nodes before therapy (*p* = 0.008). For OS, univariate analysis identified tumor size before (*p* = 0.034), after therapy (*p* = 0.041) and non-pCR (*p* = 0.041) to associate with shorter OS.

Combining the CTC results obtained here with our already published results for comprehensive CTC-analysis in this subgroup (34), Cox multivariate proportional hazard analysis additionally identified DNA excision repair protein *ERCC1*+ CTCs, associated with resistance, as an unfavorable factor for PFS (*p* = 0.026) and OS (*p* = 0.017) (Figure S1).

## Discussion

TNBC remains a subtype with a very aggressive behavior and worse outcome (1, 2). Thus, predictive biomarkers are urgently needed to stratify patients for further therapeutic options. In this context, we recently published a comprehensive CTC-analysis in three different BC subtypes before and after neoadjuvant treatment (34). Using a multi-marker gene panel including 17 different genes that target different pathways associated with stemness, EMT, resistance and survival of tumor cells, we recently demonstrated the heterogeneity of CTCs before and after therapy in these TNBC patients as compared to non-TNBC patients (34). For the group of TNBC patients, the most interesting and most important finding was the fact that ERBB2+/ERBB3+CTCs were found before and after therapy in about 20% of cases. Furthermore, EGFR+/ERBB2+/ERBB3+CTCs before and ERBB2+/ERBB3+CTCs after therapy significantly correlated with a shorter PFS (*p* = 0.01 and *p* = 0.02). Consequently, comprehensive analysis of CTCs could probably direct physicians to stratify TNBC patients for additional treatment options. The same holds true for prostate cancer related genes, especially AR, which has frequently been discussed to be a target for treatment of TNBC patients.

We here demonstrated that prostate cancer related genes expressed on CTCs in primary, non-metastatic TNBC patients were mainly found before but rarely after therapy, thus, were eliminated by the given therapy. However, *PSMA*+ CTCs before therapy significantly identified patients with worse outcome. In the context of AR inhibition, often discussed for the TNBC subgroup, this therapeutic approach might not be successful in the primary setting since we detected *AR-FL*+ CTCs together with *AR-V7*+ CTCs, associated with therapeutic failure.

AR expression in BC has mainly been studied on tissue samples resulting in a positivity rate of 10–35% (8, 19). Using immunohistochemistry for the evaluation of AR in 164 primary tumors and 83 corresponding metastases, a concordance between primary tumor and metastasis of >60% was proven (28). Consequently, the authors concluded that, if a new biopsy is performed and used for therapy selection, AR evaluation should be repeated. In another publication, this group further demonstrated that AR expression was not useful to predict the efficacy of endocrine treatment in advanced BC (40). Since metastatic biopsies are often not feasible and very invasive, CTCs as a so-called liquid biopsy have received considerable attention as a non-invasive alternative to the biopsy of metastasis and there is data suggesting that the characteristics of CTCs represent those of the metastasis better than the primary tumor (41–45). Consequently, CTCs might be more appropriate to select BC patients for AR-targeting drugs. Up to now, only very few groups have addressed the expression of prostate cancer related genes on CTCs in BC (29–33, 46). Most of these studies analyzed CTCs of metastatic HR+/HER2- BC patients for the expression of AR with a detection rate ranging from 20 to 43%, respectively (29, 31–33). Krujiff et al., further compared AR expression in primary tumor tissues and matched CTCs and observed switches from AR+ to AR-negative and *vice versa* with an overall disconcordance of 58% (32). In abiraterone/prednisone-treated postmenopausal ER+ advanced BC patients neither the analysis of biomarkers in serum, CTCs nor tumor tissue identified a subgroup a patients with significantly improved PFS, although dual expression of AR and ER in baseline CTCs were supposed to have an association with improved PFS (47). Nevertheless, these results highlight the role for AR in BC bone metastasis and suggest that inhibitory AR treatment could be successful in that subset of patients. In the mentioned studies, only AR itself but neither its splice variant nor other prostate cancer related genes were evaluated. In addition, no data were published with regard to primary BC, especially TNBC. Consequently, we can only discuss our findings with results obtained for PCA patients where CTCs have been intensively analyzed, mostly in later stages of the disease (35, 48, 49). In this regard, from the technical point of view, El-Heliebi et al., published the feasibility and utility of *in situ* padlock probe technology for the analysis of *AR-V7, AR-FL*, and *PSA* expression in combination with immunostaining (panCK and CD45) in CTCs from PCA patients (50). Furthermore, using the CellSearch system for enrichment, followed by the detection of *AR-V7* transcripts applying qPCR, adapted from the original Antonarakis et al. publication in 2014, allowed the detection of *AR-V7* and *keratin 19* (*K19*) transcripts from as low as a single *AR-V7*+/*K19*+ cell (36, 51). In the context of clinical studies, patients with CTCs expressing *AR-V7* showed worst outcome when compared to those patients harboring *AR-V7*-negative CTCs or no CTCs (52). Very recently, it was demonstrated that men with metastatic PCA who were tested positive for nuclear-localized AR-V7 protein in CTCs were likely to live longer if taxane based chemotherapy was used (53). In the PROPHECY Trial, a multicenter, prospective-blinded study of men with high-risk metastatic castration resistant PCA starting abiraterone acetate or enzalutamide treatment, the detection of *AR-V7* in CTCs by two assays was independently associated with shorter PFS and OS, concluding that such men should be offered alternative treatments (54). Based on the findings in PCA that not the AR itself but AR-V7 has been linked to resistance toward anti-AR drugs and thus, therapeutic failure, we can only speculate that AR inhibitory treatment might not be successful in non-metastatic TNBC since in two thirds of our patients with *AR*+ CTCs, *AR-V7* was also expressed. Nevertheless, although not analyzing CTCs, our findings are supported by Hickey et al., who showed that *AR-V7* protein was highly expressed in tumor tissues of a subgroup of HR-negative BCs. Moreover, they observed enzalutamide to induce *AR* and also *AR-V7* transcript expression in MDA-MB-453 cells and primary BCs. This group finally raised caution when exploring AR inhibitory treatment in women with BC and proposed the potential of AR-V7 as a predictive biomarker of anti-AR therapy response (38). We rarely found CTC-positive patients with regard to prostate cancer related genes after therapy. Thus, a decrease in CTC-positivity after therapy might also be explained by a reduction of CTC numbers under the given therapy. Due to the molecular approach used for this study, we cannot show CTC counts before and after therapy. However, we have already demonstrated that neoadjuvant therapy was able to eliminate most of the CTCs present before therapy in locally advanced BC. Interestingly, most of the residual CTCs after therapy displayed mesenchymal and/or stem cell like features (55).

Several phase II studies evaluated the effect of AR-targeting drugs in metastatic BC, especially TNBC (14, 16, 17, 56). Applying bicalutamide in AR+ but HR-negative advanced BC patients resulted in a clinical benefit rate of 19% (14) and in a multicenter single-arm trial in women with AR+, metastatic or inoperable locally advanced TNBC, the combination of abiraterone acetate plus prednisone was only beneficial for some patients with molecular apocrine tumors (16). Evaluating locally advanced or metastatic AR+ TNBC patients, enzalutamide demonstrated clinical activity and was well-tolerated, however, response rates were 25% in the intention to treat population, showing an activity in only a subset of patients (17). These preliminary studies are encouraging and understanding the AR signaling pathway harbors clinical relevance to unravel its role in TNBC pathogenesis. In this regard, AR inhibition was observed to have promising effect in preclinical studies and clinical trials with combinational approaches of AR blockade plus CDK4/6 inhibitors, PI3K inhibition, chemotherapy, and immunotherapy are currently ongoing (56).

One of our key findings was the significant correlation of *PSMA*+ CTCs with early relapse and reduced OS. Interestingly, using the same method for the detection of *PSMA*+ CTCs, *PSMA* transcript declines appeared to be associated with concurrent decreases in serum PSA, thus, sequential CTC sampling was proposed to provide a non-invasive response assessment to systemic treatment for metastatic castration-resistant PCA (57).

PSMA expression was detected in endothelial cells of the neovasculature, but not in adjacent normal endothelium, thus, its expression has already been studied in a variety of cancer tissues, including TNBC. In this context, Morgenroth et al. recently identified PSMA as potential target for radio-ligand therapy in TNBC MDA-MB231 cells (11). Kasoha et al., observed PSMA to be expressed in the neovasculature of breast tumors and its distant metastases. Interestingly, the ^68^Ga-PSMA tracer was strongly uptaken in the bone metastases of a metastatic BC patients, elucidating PSMA as a therapeutic vascular target (10). In the management of PCA, PSMA has already become an attractive target for oncological imaging and radionuclide therapy since its expression persisted in a high percentage of these patients, confirmed by positron emission tomography/computer tomography (58). These findings supported the use of imaging for diagnostic purposes as compared to the assessment of blood-based PSA values (59–63). For PCA, radioligand therapy using 177Lu-PSMA-617 was shown to be safe with a low toxicity profile and PSMA-11-derived dual-labeled PSMA inhibitors for preoperative imaging and guided surgery were feasible to detect PSMA-specific PCA lesions (64, 65).

### Conclusion and Limitation of the Study

To validate the feasibility of our blood-based approach, a comparison of blood and tissue would have been necessary. However, before therapy, at least three tissue biopsies are taken for diagnostic purposes while the remaining tissue is kept as a so-called “back-up” for repeating analysis or additional analyses in case of relapse. After therapy, the same holds true since neoadjuvant chemotherapy results in tumor shrinkage in most cases, reducing the chance of tissue analysis for other purposes than diagnostics. In addition, a comparison of CTC characteristics on the mRNA level and CTC characteristics on the protein level would have been interesting. However, the CTC isolation method used in this study is not suitable for protein expression analysis, making a direct comparison of matched CTC samples for RNA and protein analysis not feasible. It is to mention that all currently available CTC isolation methods, including the one used for the current study, do not capture the entirety of CTCs. However, using positive immunomagnetic selection targeting EpCAM, HER2, and EGFR improved and optimized the enrichment of tumor stem cell and EMT like CTC compared to cell capturing with anti-EpCAM alone in different tumor entities (66–68).

Nevertheless, to the best of our knowledge, this is the first study, comprehensively analyzing some prostate cancer related genes in CTCs of a defined primary, non-metastatic TNBC subgroup before and after therapy. Although expressed in a minority of patients, *PSMA*+ CTCs significantly identified patients with worse outcome and could serve as a new predictive marker in this BC subgroup, probably in combination with 68Ga-PSMA imaging or even as target for treatment. Furthermore, in the context of AR inhibition, our findings demonstrate that this treatment option might not be successful in the primary setting of TNBC since we identified *AR-FL*+ CTCs together with *AR-V7*+ CTCs, associated with therapeutic failure. However, these findings carefully have to be evaluated in further clinical studies.

## Materials and Methods

### Patient Characteristics

The study was conducted at the Department of Gynecology and Obstetrics, at the University Hospital of Essen, in Germany. In total, 41 early TNBC patients (before therapy: *n* = 41, matched samples after therapy *n* = 26), diagnosed between January 2013 and August 2018, were enrolled. All patients presented with first diagnosis of TNBC in our clinic, were non-metastatic and had not been treated before. Blood was obtained after written informed consent from all subjects using protocols approved by the clinical ethic committee of the University Hospital Essen (05/2856). Patient characteristics are documented in Table 1.

### Eligibility Criteria and Response Criteria

The eligibility criteria were as follows: histologically proven BC, no severe uncontrolled comorbidities or medical conditions, and no further malignancies at present or in the patient history. Blood was drawn at primary diagnosis and after NACT. Completion of NACT (*n* = 39) or adjuvant treatment (*n* = 2) (anthracyclines, taxanes, cyclophosphamide, carbo- and cisplatin, gemcitabine; Table S1) were applied according to current guidelines as well as radiotherapy (3). Two patients received the PARP-inhibitor Olaparib in a clinical trial (GeparOla). For each of the 41 patients, the tumor type, TNM-staging, grading and Ki67 were assessed in the Institute of Pathology, at the University Hospital Essen as part of the West German Comprehensive Cancer Center. Pathological response to therapy was defined according to the grading system of Sinn et al., 1994 (69): 0 = no effect; 1 = resorption and tumor sclerosis; 2 = minimal residual invasive tumor (<0.5 cm); 3 = residual non-invasive tumor only, ductal carcinoma *in situ* (DCIS); 4 = no tumor detectable. pCR was defined as regression 4 according to Sinn, no evidence of residual invasive cancer and DCIS, both, in breast and axilla; pathological partial response (pPR) was defined as regression 1–3 according to Sinn (69).

### Sampling of Blood

2 x 5 ml EDTA blood were collected for CTC isolation in S-Monovettes^®^ (Sarstedt AG & Co., Germany). Samples were stored at 4°C and processed not later than 4 h after blood withdrawal.

### Enrichment of Circulating Tumor Cells, mRNA Isolation, and Reverse Transcription

Positive immunomagnetic selection targeting EpCAM, EGFR, and HER2 (AdnaTest EMT-2/StemCell Select™, QIAGEN GmbH, Hilden, Germany) was employed for CTC isolation from 2 × 5 ml blood. The method has been described in detail elsewhere (70). mRNA was isolated by oligo(dT)_25_-beads and was reverse transcribed (AdnaTest *EMT-2/*StemCell Detect™, QIAGEN GmbH, Hilden, Germany). The final reaction volume of 40 μl cDNA was stored at −20°C.

### Quantitative PCR

The multimarker RT-qPCR AdnaTest *ProstateCancerPanel AR-V7* detecting *CD45 (PTPRC), PSA (KLK3), PSMA (FOLH1), full-length AR (AR-FL), AR splice variant seven* (*AR-V7)*, and *GAPDH* (QIAGEN GmbH, Hilden, Germany) has been described in detail recently (71–73). The primer set to detect *AR-FL* does not detect the *AR-V7* transcript. The method requires transcript-specific pre-amplification of 6.25 μl cDNA using the 2xMultiplex PCR Master Mix (QIAGEN GmbH, Hilden, Germany) with 18 PCR cycles. PCR was performed as follows: denaturation for 5 min at 95°C, followed by 18 cycles of 30 s at 95°C, 90 s at 60°C, and 90 s at 72°C. Preamplified cDNA (3 μl; 1:10 diluted) was analyzed in duplicates for one of the six transcripts in a reaction volume with miRCURY SYBR Green MasterMix (QIAGEN GmbH, Hilden, Germany) and ROX Reference Dye (0.75 μl; QIAGEN GmbH, Hilden, Germany) of in total 15 μl. RT-qPCR was performed with the StepOnePlus™ (Thermo Fisher Scientific, Waltham, USA) real-time system as follows: PCR activation for 10 min at 95°C, followed by 35 cycles 10 s at 95°C, 10 s at 60°C, and 10 s at 78°C. In addition to fluorescence readout at 78°C in each cycle, melting curves were obtained.

### Data Evaluation

CTC isolation was conducted in duplicate from 2 × 5 ml blood for each patient sample. cDNA was analyzed separately from these duplicates. The fluorescence threshold of 0.48 was employed for all transcripts (programmed with StepOne Software v2.3) and defined the PCR cycle used for transcript quantification. CTC expression data was normalized to data of healthy donor controls (*n* = 14) using individual cut off values for each gene (raw data shown in Table S2). *GAPDH* not exclusively expressed in CTCs but also in the 100–200 contaminating leukocytes was normalized to the leukocyte-specific transcript *PTPRC* (ΔΔCq = [Cutoff(gene)-Sample Cq(gene)]-[Cutoff(*PTPRC*)-Sample Cq(*PTPRC*)]. The transcripts *PSA, PSMA, AR-FL, AR-V7* were independent of a growing number of leukocytes, thus, the ΔCq value was calculated as follows: ΔCq = [Cutoff(gene)-Sample Cq(gene)]. Only positive Δ(Δ)Cq values were considered as evaluable signals and signals were analyzed binary to be interpreted as overexpression yes/no results. Results of primer that showed Cq values below 35 in the negative control and results of amplicons with the wrong melt temperature [ΔTm (positive control – sample) >2°C] and [Tm <76.6°C] were excluded. We evaluated a sample to be positive for one transcript, if at least one of the two sample duplicates showed a Cq value below the cut-off. Cq values of all patient samples and healthy donors are listed in Table S2.

### Statistical Analysis

Statistical analysis was performed using R (version 3.6.1) with R packages shiny, hmisc, ggplot2, survival, broom, and dplyr. Survival intervals were screened from the time of first diagnosis until the date of recurrence (PFS) or death (OS) and calculated with Kaplan-Meier estimator (Log-rank test). In this cohort, recurrence was supposed in all deceased patients (*n* = 2) who had no documented BC associated death. In addition, a univariate and multivariate Cox proportional hazard analysis was conducted to confirm the Kaplan Meier findings and to identify factor dependencies. *P* < 0.05 were considered to indicate a statistically significant difference. Diagrams were computed with the R script mentioned or with Microsoft Excel (Microsoft Corporation, Redmond, WA, USA).

## Data Availability Statement

All datasets presented in this study are included in the article/Supplementary Material.

## Ethics Statement

The studies involving human participants were reviewed and approved by clinical ethic committee of the University Hospital Essen (05/2856). The patients/participants provided their written informed consent to participate in this study.

## Author Contributions

SK-B and RK supervised the study. SK-B developed the concept and design of the study. SH established and developed the method. CK established, validated the method in the Department of Gynecology and Obstetrics, University Hospital Essen, and wrote sections of the manuscript. OH and A-KB recruited the patients. SK-B, CK, OH, and A-KB collected the experimental and clinical data. SK-B, CK, and SH evaluated, analyzed the data, reviewed, and edited the manuscript. CK and SH performed the statistical analysis and visualized the results. SK-B and A-KB wrote the first draft of the manuscript. All authors contributed to manuscript revision, read, and approved the submitted version.

## Conflict of Interest

SK-B is a consultant for QIAGEN and has received honoraria from Novartis. CK received support for travel expenses from QIAGEN. OH received honoraria from Roche, Amgen, Pfizer, MSD, and Novartis. SH is an employee at QIAGEN. RK has received honoraria/is part of the advisory board from/at Tesaro, Astra-Zeneca, Medtronic in the last 3 years, and council of IGCS, president of SERGS and proctored and presented for Intuitive Surgical. A-KB received honoraria from Roche.

## Abbreviations

AR: androgen receptor
AR-FL: AR-full length
HGNC ID:644
AR-V7: androgen receptor variant seven
BC: breast cancer
CTCs: circulating tumor cells
ER: estrogen receptor
HR: hormonal receptors
LAR: luminal androgen receptor
NACT: neoadjuvant chemotherapy
OS: overall survival
PCA: prostate cancer
pCR: pathological complete response
PFS: progression free survival
pPR: pathological partial response
PR: progesterone receptor
PSA: prostate specific antigen
HGNC: KLK3: ID:6364
PSMA: prostate specific membrane antigen
HGNC: FOLH1
ID:3788
pts: patients
TNBC: triple-negative breast cancer.
