# Supplementary material for: Circulating Tumor Cells Expressing the Prostate Specific Membrane Antigen (PSMA) Indicate Worse Outcome in Primary, Non-Metastatic Triple-Negative Breast Cancer
Source: Front Oncol. 2020 Sep 3;10:1658. doi: 10.3389/fonc.2020.01658 (PMC7497312; doi:10.3389/fonc.2020.01658)
Supplement: Supplementary file 1 [file Data_Sheet_1.PDF]

Supplementary Material

1 Supplementary Figures

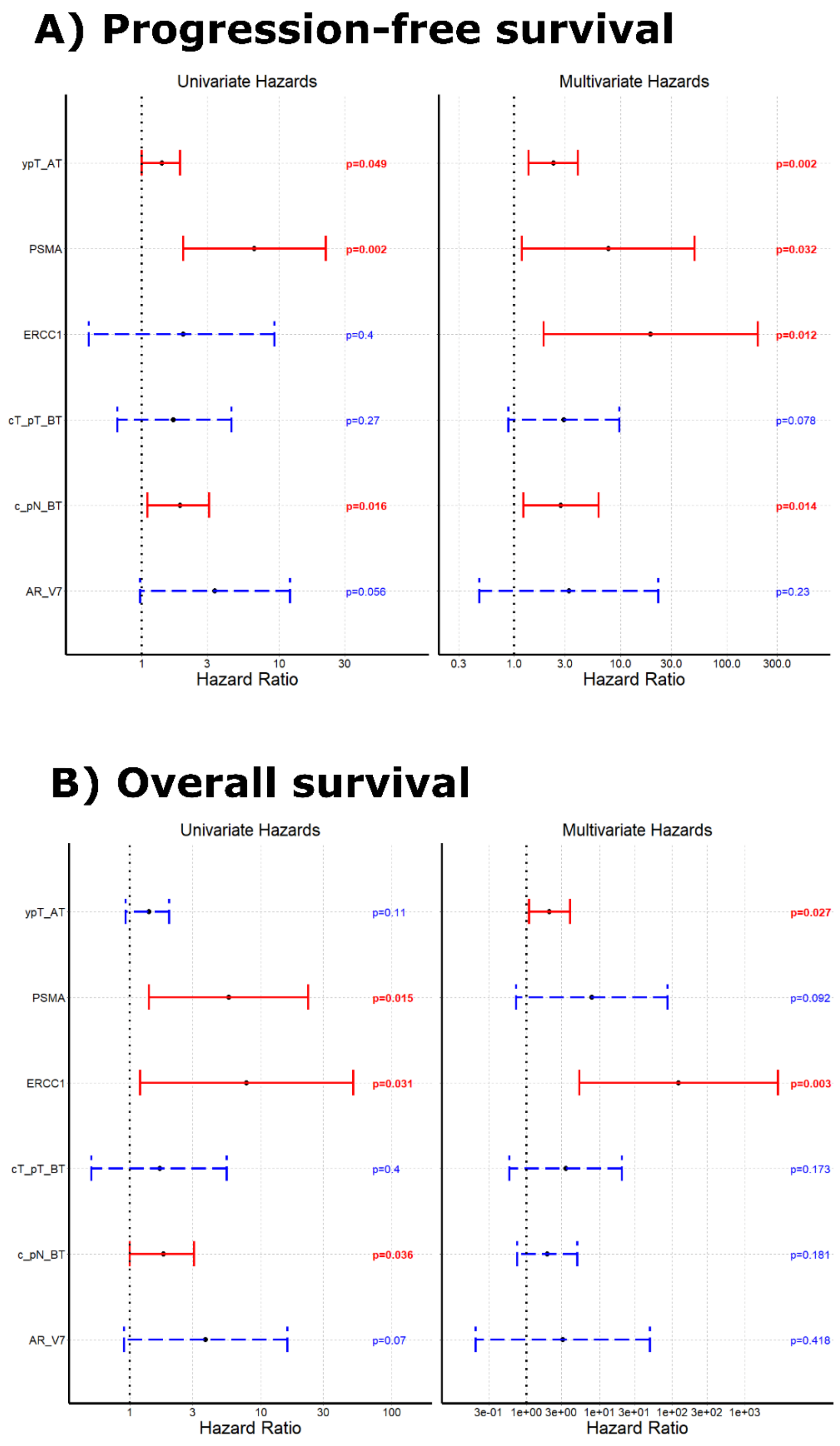

Supplementary Figure 1. Univariate and multivariate Cox proportional hazard analysis regarding PFS (A) and OS (B) for patients with *PSMA*+, *ARV7*+ and *ERC*C1+ CTCs. ypT_AT: tumor size after therapy, cT_pT_BT: tumor size before therapy, c_pN_BT: lymph node status before therapy

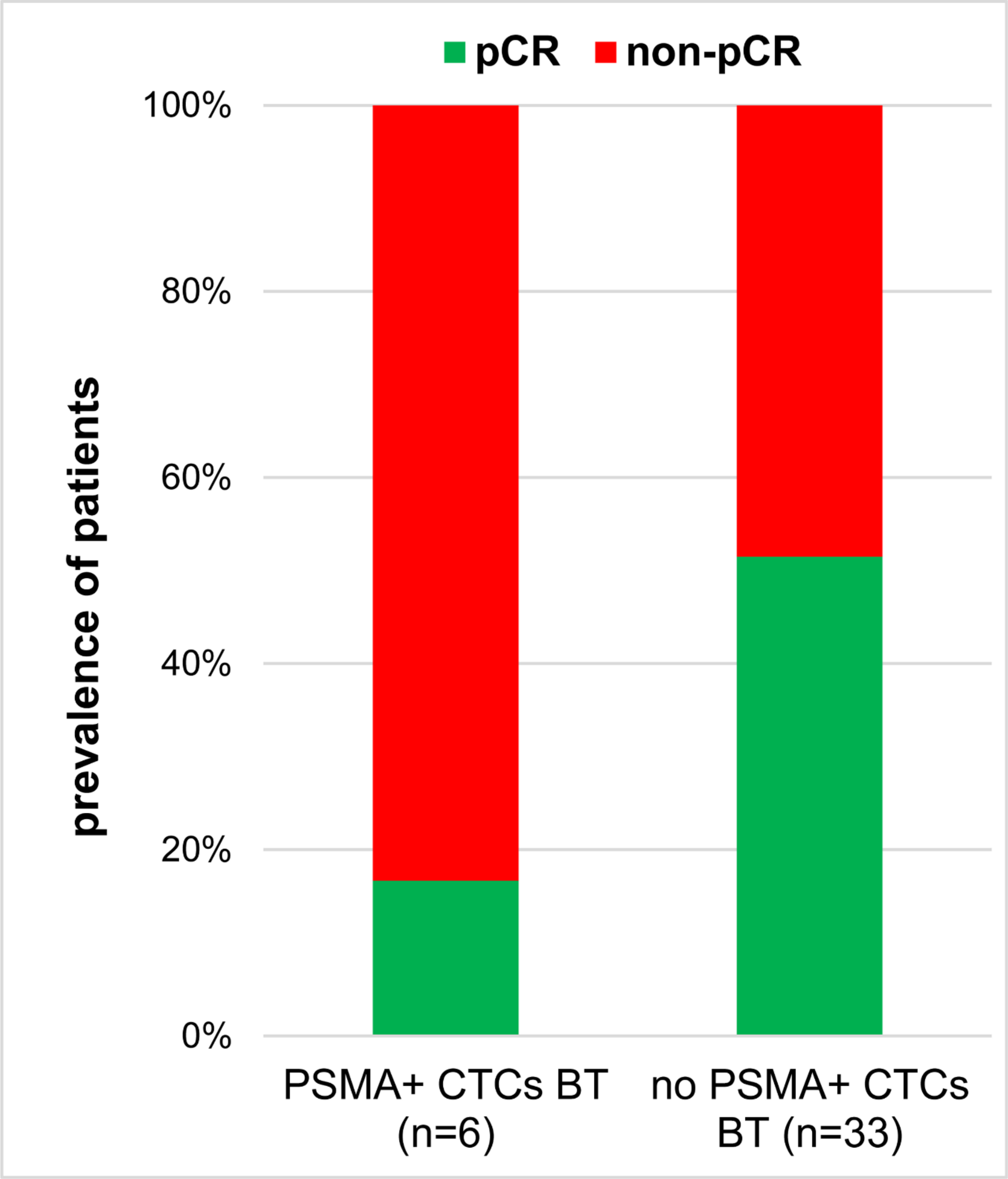

	PSMA + CTCs BT	no *PSMA* + CTCs BT
**non-pCR**	5	16
**pCR**	1	17

Supplementary Figure 2. Prevalence of patients with *PSMA*+ CTCs showing non-pCR.

Supplementary Table 1. Systemic therapy regiments. Legend: PD = progressive disease

Therapeutic regimens	Number of patients
Anthracycline/ Cyclophosphamid/ Taxane	21
Anthracycline/ Cyclophosphamid/ Taxane/ Carboplatin	3
Anthracycline/ Cyclophosphamid/ Taxane/ PARP-Inhibitor	2
Taxane/ Carboplatin	2
Nab-Paclitaxel/ Gemcitabin	2
Anthracycline/ Taxane/ Carboplatin	8
Cisplatin/ Gemcitabin	1
Paclitaxel/ Gemcitabin/ Anthracycline/ Cyclophosphamid	1
Anthracycline	1
Anthracycline/ Cyclophosphamid/ Taxane/ PD: Carboplatin/ Gemcitabin / PD: Xeloda/ Avastin	1

Supplementary Table 2. Raw data. Cq values of all patient samples [in duplicate (/1 and /2); n=41 before and n=26 after therapy] and healthy donors (n=14). Overexpression signals of the prostate-cancer related genes for all patient samples calculated by ΔCq = [Cutoff(gene)-Sample Cq(gene)]. Positive ΔCq were regarded as evaluable signals and signals were only analyzed binary (1=overexpression signal; 0= no overexpression signal). Relapse data for progression-free survival (PFS) and overall survival (OS) for all 41 patients analyzed before therapy.

		C_q values						overexpression signals				PFS		OS	
cohort	patient sample	*CD45*	*GAPDH*	*PSA*	*PSMA*	*AR-FL*	*AR-V7*	*PSA*	*PSMA*	*AR-FL*	*AR-V7*	Progression	PFS time [months]	Death	OS time [months]
Healthy donor (HD) controls	HD1	27,374	29,08	35,00	35,00	35,00	35,00								
	HD2	26,96	30,06	35,00	35,00	35,00	35,00								
	HD3	25,17	25,52	35,00	35,00	35,00	35,00								
	HD4	25,97	26,25	35,00	35,00	35,00	35,00								
	HD5	26,97	28,46	35,00	35,00	35,00	35,00								
	HD6	27,31	29,81	35,00	35,00	35,00	35,00								
	HD7	24,24	28,26	35,00	35,00	35,00	35,00								
	HD8	26,29	29,07	35,00	35,00	35,00	35,00								

	HD9	27,58	26,57	35,00	35,00	35,00	35,00								
	HD10	24,36	27,75	35,00	35,00	35,00	35,00								
	HD11	24,49	27,78	35,00	35,00	35,00	35,00								
	HD12	27,19	29,39	35,00	35,00	35,00	35,00								
	HD13	29,94	34,76	35,00	35,00	34,32	35,00								
	HD14	27,71	30,72	35,00	35,00	35,00	35,00								
	Mean C_q value of HDs	26,54	28,82	35,00	35,00	34,95	35,00								
	Standard deviation of HDs	1,58	2,27	0,00	0,00	0,18	0,00								
	Cut off value	24,96	24,28	35,00	35,00	35,00	35,00								
	Specificity	78%	100%	100%	100%	100%	100%								
**TNBC patients before therapy**	976/1	22,70	21,87	34,17	35,00	35,00	35,00	1	0	0	0	0	77	0	77
	976/2	22,16	22,05	35,00	35,00	35,00	35,00	0	0	0	0				
	987/1	28,62	32,29	35,00	33,36	35,00	35,00	0	1	0	0	1	19	0	68
	987/2	32,25	34,49	35,00	35,00	35,00	35,00	0	0	0	0				
	992/1	35,00	31,31	35,00	35,00	35,00	35,00	0	0	0	0	0	53	0	53
	992/2	29,23	31,10	35,00	35,00	35,00	35,00	0	0	0	0				
	994/1	28,58	31,40	35,00	35,00	35,00	35,00	0	0	0	0	0	72	0	72
	994/2	31,20	31,79	35,00	35,00	35,00	35,00	0	0	0	0				
	998/1	28,45	31,46	35,00	35,00	35,00	35,00	0	0	0	0	0	74	0	74
	998/2	28,13	31,47	35,00	35,00	35,00	35,00	0	0	0	0				
	1001/1	24,90	27,78	35,00	35,00	35,00	35,00	0	0	0	0	0	50	0	50
	1001/2	24,67	27,80	33,03	35,00	35,00	35,00	1	0	0	0				
	1008/1	28,01	31,09	35,00	35,00	35,00	35,00	0	0	0	0	0	55	0	55
	1008/2	27,54	29,69	35,00	35,00	35,00	35,00	0	0	0	0				

1132/1	26,14	29,30	35,00	35,00	35,00	35,00	0	0	0	0	0	61	0	61
1132/2	25,82	29,04	35,00	35,00	35,00	35,00	0	0	0	0				
1717/1	30,37	32,46	35,00	35,00	35,00	34,93	0	0	0	0	0	57	0	57
1741/1	25,55	26,21	35,00	35,00	35,00	35,00	0	0	0	0	1	9	1	21
1741/2	24,95	25,65	35,00	35,00	35,00	35,00	0	0	0	0				
1765/1	35,00	30,19	35,00	33,65	35,00	35,00	0	1	0	0	1	14	1	20
1765/2	35,00	29,52	35,00	35,00	35,00	35,00	0	0	0	0				
1766/1	35,00	31,21	33,84	33,39	31,00	34,16	1	1	1	0	0	46	0	46
1766/2	35,00	30,14	35,00	33,97	26,65	34,99	0	1	1	0				
1768/1	32,13	35,00	35,00	33,88	31,97	34,45	0	1	1	1	1	16	1	33
1768/2	25,82	29,61	Dimer	32,95	Dimer	34,74	0	1	0	1				
1769/1	35,00	30,78	35,00	33,39	32,92	33,20	0	1	1	1	1	10	1	29
1778/1	21,59	20,22	35,00	35,00	35,00	35,00	0	0	0	0	0	56	0	56
1778/2	21,20	20,01	35,00	35,00	35,00	35,00	0	0	0	0				
1800/1	21,03	22,22	35,00	35,00	35,00	35,00	0	0	0	0	0	42	0	42
1800/2	21,78	23,00	35,00	35,00	35,00	35,00	0	0	0	0				
1808/1	21,30	20,78	35,00	35,00	35,00	35,00	0	0	0	0	0	52	0	52
1808/2	35,00	35,00	35,00	35,00	35,00	35,00	0	0	0	0				
1810/1	20,32	19,34	35,00	35,00	35,00	35,00	0	0	0	0	0	48	0	48
1810/2	20,23	19,49	35,00	35,00	35,00	35,00	0	0	0	0				
1811/1	21,04	19,95	35,00	35,00	35,00	35,00	0	0	0	0	0	45	0	45
1811/2	21,31	20,52	Dimer	35,00	35,00	35,00	0	0	0	0				
1814/1	21,94	20,85	35,00	35,00	35,00	35,00	0	0	0	0	0	52	0	52

1814/2	21,33	19,77	35,00	35,00	35,00	35,00	0	0	0	0				
1821/1	20,98	21,81	35,00	35,00	35,00	35,00	0	0	0	0	0	51	0	51
1821/2	21,24	22,11	35,00	35,00	35,00	35,00	0	0	0	0				
1822/1	24,12	25,94	34,63	35,00	35,00	35,00	1	0	0	0	0	28	0	28
1822/2	22,98	27,01	35,00	35,00	35,00	35,00	0	0	0	0				
1830/1	20,37	19,54	35,00	35,00	35,00	35,00	0	0	0	0	0	30	0	30
1830/2	21,70	20,94	35,00	35,00	35,00	35,00	0	0	0	0				
1849/1	20,23	23,97	35,00	35,00	35,00	35,00	0	0	0	0	1	13	1	19
1849/2	19,43	23,43	35,00	35,00	35,00	35,00	0	0	0	0				
1854/1	20,38	21,16	35,00	35,00	35,00	25,77	0	0	0	1	1	34	1	34
1854/2	20,30	20,94	Dimer	34,37	27,31	33,32	0	1	1	1				
1856/1	22,67	22,05	35,00	35,00	25,90	27,25	0	0	1	1	0	48	0	48
1856/2	22,73	21,49	35,00	35,00	22,96	26,91	0	0	1	1				
1939/1	21,86	20,01	35,00	35,00	22,92	26,01	0	0	1	1	0	18	0	18
1939/2	22,40	20,95	35,00	35,00	26,25	35,00	0	0	1	0				
1948/1	22,97	21,52	35,00	35,00	35,00	35,00	0	0	0	0	0	22	0	22
1948/2	24,81	21,45	35,00	35,00	22,23	35,00	0	0	1	0				
1966/1	20,48	18,55	35,00	35,00	35,00	35,00	0	0	0	0	1	19	1	38
1966/2	21,10	18,47	35,00	35,00	35,00	35,00	0	0	0	0				
1967/1	20,95	19,99	35,00	35,00	35,00	35,00	0	0	0	0	1	18	0	35
1967/2	21,55	20,04	33,55	35,00	23,94	28,14	1	0	1	1				
1970/1	23,46	22,33	35,00	35,00	25,79	35,00	0	0	1	0	0	28	0	28
1970/2	23,37	21,75	35,00	35,00	35,00	35,00	0	0	0	0				

	1974/1	21,36	19,95	35,00	35,00	23,96	35,00	0	0	1	0	0	18	0	18
	1974/2	20,81	19,41	Dimer	Dimer	25,03	26,04	0	0	1	1				
	1986/1	19,78	18,20	35,00	35,00	25,57	26,96	0	0	1	1	0	18	0	18
	1986/2	19,48	18,02	35,00	35,00	26,31	25,66	0	0	1	1				
	2091/1	24,41	26,78	35,00	35,00	35,00	35,00	0	0	0	0	0	36	0	36
	2091/2	23,98	25,57	35,00	35,00	35,00	35,00	0	0	0	0				
	2094/1	27,80	28,39	35,00	35,00	35,00	35,00	0	0	0	0	1	22	0	32
	2094/2	35,00	26,01	35,00	35,00	35,00	35,00	0	0	0	0				
	2096/1	26,24	22,67	35,00	35,00	35,00	35,00	0	0	0	0	0	31	0	31
	2096/2	25,85	21,53	35,00	35,00	35,00	35,00	0	0	0	0				
	2100/1	25,94	22,78	35,00	35,00	35,00	35,00	0	0	0	0	1	13	1	31
	2100/2	24,12	24,78	35,00	35,00	35,00	35,00	0	0	0	0				
	2105/1	22,14	23,64	35,00	35,00	35,00	35,00	0	0	0	0	0	29	0	29
	2105/2	21,27	23,22	35,00	35,00	35,00	35,00	0	0	0	0				
	2132/1	24,03	25,48	35,00	35,00	35,00	35,00	0	0	0	0	0	26	0	26
	2132/2	22,82	24,36	35,00	35,00	35,00	35,00	0	0	0	0				
	2150/1	21,24	22,11	35,00	35,00	35,00	35,00	0	0	0	0	0	21	0	21
	2150/2	21,50	22,41	35,00	35,00	35,00	35,00	0	0	0	0				
	2183/1	22,16	22,20	35,00	35,00	35,00	35,00	0	0	0	0	0	15	0	15
	2183/2	21,73	22,97	35,00	35,00	35,00	35,00	0	0	0	0				
**TNBC patients**	1071/1	28,44	35,00	35,00	35,00	35,00	35,00	0	0	0	0				
	1071/2	25,10	26,68	35,00	35,00	35,00	35,00	0	0	0	0				
	1276/1	35,00	27,87	Dimer	35,00	35,00	35,00	0	0	0	0				

	1276/2	25,29	23,46	35,00	35,00	35,00	35,00	0	0	0	0
	1681/1	27,56	30,94	35,00	35,00	35,00	35,00	0	0	0	0
	1681/2	29,66	30,12	35,00	35,00	35,00	35,00	0	0	0	0
	1826/1	25,33	24,53	35,00	35,00	35,00	35,00	0	0	0	0
	1826/2	24,14	25,94	35,00	35,00	35,00	35,00	0	0	0	0
	1861/1	18,95	22,61	35,00	35,00	35,00	35,00	0	0	0	0
	1861/2	19,05	22,28	35,00	35,00	35,00	35,00	0	0	0	0
	1871/1	23,51	24,04	35,00	35,00	35,00	35,00	0	0	0	0
	1871/2	24,31	24,44	35,00	35,00	35,00	35,00	0	0	0	0
	1890/1	24,97	35,00	35,00	35,00	35,00	35,00	0	0	0	0
	1890/2	27,15	35,00	35,00	35,00	35,00	35,00	0	0	0	0
	1893/1	25,02	24,63	35,00	35,00	35,00	35,00	0	0	0	0
	1893/2	26,27	25,56	35,00	35,00	35,00	35,00	0	0	0	0
	1901/1	27,45	35,00	35,00	35,00	Dimer	35,00	0	0	0	0
	1901/2	24,80	27,71	Dimer	Dimer	Dimer	35,00	0	0	0	0
	1907/1	26,62	27,92	35,00	35,00	35,00	35,00	0	0	0	0
	1907/2	27,04	29,51	Dimer	35,00	35,00	35,00	0	0	0	0
	1908/1	25,87	26,24	35,00	35,00	35,00	35,00	0	0	0	0
	1908/2	25,70	25,46	35,00	35,00	35,00	35,00	0	0	0	0
	1913/1	23,26	24,75	35,00	35,00	35,00	35,00	0	0	0	0
	1913/2	24,78	26,15	35,00	35,00	35,00	35,00	0	0	0	0
	1920/1	20,96	21,86	35,00	35,00	35,00	35,00	0	0	0	0

	1920/2	20,38	21,38	35,00	35,00	35,00	35,00	0	0	0	0
	1927/1	24,36	26,87	35,00	35,00	35,00	35,00	0	0	0	0
	1927/2	24,17	24,05	35,00	35,00	35,00	35,00	0	0	0	0
	1940/1	23,29	24,17	35,00	35,00	35,00	35,00	0	0	0	0
	1940/2	23,73	24,54	35,00	35,00	35,00	35,00	0	0	0	0
	1956/1	21,25	22,97	Dimer	25,91	24,16	27,26	0	1	1	1
	1956/2	23,29	22,90	35,00	35,00	35,00	35,00	0	0	0	0
	2023/1	25,36	23,22	35,00	35,00	35,00	35,00	0	0	0	0
	2023/2	26,03	22,26	35,00	35,00	35,00	35,00	0	0	0	0
	2051/1	24,38	24,09	35,00	35,00	35,00	35,00	0	0	0	0
	2051/2	23,68	24,02	35,00	35,00	35,00	35,00	0	0	0	0
	2058/1	21,98	23,83	35,00	35,00	35,00	35,00	0	0	0	0
	2058/2	22,00	23,41	35,00	35,00	35,00	35,00	0	0	0	0
	2061/1	25,33	25,59	35,00	35,00	35,00	35,00	0	0	0	0
	2061/2	25,71	35,00	35,00	35,00	35,00	35,00	0	0	0	0
	2163/1	35,00	21,83	35,00	35,00	35,00	35,00	0	0	0	0
	2163/2	25,36	21,59	35,00	35,00	35,00	35,00	0	0	0	0
	2167/1	25,73	22,96	35,00	35,00	35,00	35,00	0	0	0	0
	2167/2	26,42	21,42	35,00	35,00	35,00	35,00	0	0	0	0
	2172/1	24,36	23,34	35,00	35,00	35,00	35,00	0	0	0	0
	2172/2	25,72	23,80	35,00	35,00	35,00	35,00	0	0	0	0
	2191/1	23,82	22,29	35,00	35,00	35,00	35,00	0	0	0	0
	2191/2	24,26	22,96	35,00	35,00	35,00	35,00	0	0	0	0

	2206/1	25,87	25,41	35,00	35,00	35,00	35,00	0	0	0	0
	2206/2	24,59	24,56	35,00	35,00	35,00	35,00	0	0	0	0
	2249/1	35,00	27,43	35,00	35,00	35,00	35,00	0	0	0	0
	2249/2	26,65	27,20	35,00	35,00	35,00	35,00	0	0	0	0

Supplementary Table 3. Hazard Ratios and Confidence Intervals for the Cox univariate and multivariate proportional hazard analysis regarding PFS and OS (Figure 3). ypT_AT: tumor size after therapy, cT_pT_BT: tumor size before therapy, c_pN_BT: lymph node status before therapy

	**correlation to overall survival**					
	**univariate**			**multivariate**		
	**HR**	**lower CI**	**upper CI**	**HR**	**lower CI**	**upper CI**
**ypT_AT**	1.4	1	2	1.76	0.7	4.42
PSMA	5.7	1.4	23	2.92	0.36	24.36
**non-pCR**	8.7	1.1	70	6.13	0.41	92.77
**cT_pT_BT**	2.9	1.1	7.7	0.63	0.09	4.69
**c_pN_BT**	1.6	0.96	2.7	1.96	0.82	4.66
AR-V7	3.8	0.9	16	1.33	0.12	5.13

	**correlation to disease-free survival**					
	**univariate**			**multivariate**		
	**HR**	**lower CI**	**upper CI**	**HR**	**lower CI**	**upper CI**
**ypT_AT**	1.5	1.1	2	2.61	0.95	7.16
PSMA	6.6	2	22	2.42	0.46	12.76
**non-pCR**	15	1.9	110	27.09	1.15	638.04
**cT_pT_BT**	2.4	1	5.5	0.63	0.12	3.28
**c_pN_BT**	1.7	1.1	2.8	3.3	1.36	8.05
AR-V7	3.4	0.97	12	4.3	0.55	33.35
